# An Experimental Study on the Precision Abrasive Machining Process of Hard and Brittle Materials with Ultraviolet-Resin Bond Diamond Abrasive Tools

**DOI:** 10.3390/ma12010125

**Published:** 2019-01-02

**Authors:** Lei Guo, Xinrong Zhang, Shibin Chen, Jizhuang Hui

**Affiliations:** 1 Key Laboratory of Road Construction Technology and Equipment, Chang’an University, South 2nd Ring, Xi’an 710064, Shannxi, China; zxrong@chd.edu.cn (X.Z.); sbchen@chd.edu.cn (S.C.); huijz@chd.edu.cn (J.H.); 2 Shaanxi Fast Auto Drive Engineering Technology Research Center, West Avenue, Xi’an 710119, Shannxi, China

**Keywords:** abrasive machining, sapphire substrate, resin bond

## Abstract

Ultraviolet-curable resin was introduced as a bonding agent into the fabrication process of precision abrasive machining tools in this study, aiming to deliver a rapid, flexible, economical, and environment-friendly additive manufacturing process to replace the hot press and sintering process with thermal-curable resin. A laboratory manufacturing process was established to develop an ultraviolet-curable resin bond diamond lapping plate, the machining performance of which on the ceramic workpiece was examined through a series of comparative experiments with slurry-based iron plate lapping. The machined surface roughness and weight loss of the workpieces were periodically recorded to evaluate the surface finish quality and the material removal rate. The promising results in terms of a 12% improvement in surface roughness and 25% reduction in material removal rate were obtained from the ultraviolet-curable resin plate-involved lapping process. A summarized hypothesis was drawn to describe the dynamically-balanced state of the hybrid precision abrasive machining process integrated both the two-body and three-body abrasion mode.

## 1. Introduction

Hard and brittle materials in the forms of silicon, sapphire, glass, and different types of ceramics have gradually become one of the most broadly used materials in the modern industry. Thanks to their superior material properties in chemical, physical, optical, and electronic characteristics, hard and brittle materials can be utilized in various fields from the screen of cell phones to the optics cavity of laser gyros. However, the machining of this material is still challenging due to their extreme hardness, brittleness, and chemical stability. According to the specific application, the aim of hard and brittle material machining is not just to remove the material efficiently, but also to ensure a desirable surface quality in terms of surface flatness, surface roughness, and surface integrity. These characteristics are mainly determined by the material removal mechanism of the precision machining processes, like lapping and polishing, thereby the role of the machining tools that directly contact the materials in these machining processes significantly affect the output quality of the process.

Lapping plates fabricated with metals, such as cast iron (Fe) and copper (Cu), have been broadly used for semiconductor material abrasive machining processes. These metal plates are capable of providing a relatively higher material removal rate, and they are also easily manufactured economically. After lapping with these hard plates, a softer tool, like a tin (Sn)/lead (Pb) plate or metal-resin composite plate, is utilized in the polishing process to perform atomic-level material removal, and a high-quality surface finish can be obtained meanwhile as a result [1]. However, in addition to the machining tool as introduced, lapping and polishing processes are also primarily affected by the involved abrasive slurry, which is a mixture of the abrasive grains and liquid carrier that can be either oil-based or water-based. During the machining process, the abrasive grains from the slurry are rolling between the workpiece and base plate and the material is mainly removed in three-body abrasion mode. On the other hand, in some case of the lapping process, the abrasive grains are fixed on the plate through the fabrication process, so the material is mostly removed from the workpiece in two-body abrasion. According to Kim et al. [2], a relatively higher material removal rate can be obtained through two-body abrasion lapping, while a dense abrasive slurry is needed for three-body abrasion to achieve the same rate. As a result, both the process waste and cost increased significantly in the slurry-based lapping process, and environmental pollution could be another potential issue.

Recently, researchers focused their attention toward the two-body abrasion mode in the fixed abrasive lapping plate [3]. However, considering the surface finish quality, the two-body abrasion produces a rougher surface than three-body abrasion. For this reason, researchers around the world started to study the possibility of combining the two-body and three-body abrasion modes to integrate their advantages in machining efficiency and surface finish. Luo et al. [4] tried to develop a so-called semi-fixed abrasive tool with sol-gel technology to form a softer bond between the abrasive grains and bonding agent. During the machining process, the fall-off semi-fixed abrasive grains could work in three-body abrasion mode with the fixed grains working in two-body abrasion mode. The fabrication process was based on the cross-linking reaction of the sodium alginate (AGS) at certain conditions. Therefore, the reaction completion and process parameter control could potentially limit the application of this technology. Based on the conventional thermal press and sintering process, Pyun et al. [5] fabricated a high-performance copper-resin plate for sapphire machining to combine the two-body and three-body abrasion mode, and examined the influence of different amounts of curing agent as a function of resin weight. The interface between the Cu and resin and the hardness of the lapping plate were found to be the primary factor affecting the material removal, and thereby caused the temporary two-body abrasive transformed to three-body abrasive. All the investigations introduced above have shown promising results in terms of the material removal rate and nanoscale surface roughness on the machining of hard and brittle materials.

In this paper, we proposed a new abrasive machining tool fabricated with ultraviolet-curable resin and diamond abrasive grains. Resin bond is one of the most widely used bonding agents in the manufacturing of abrasive tools including grinding wheel, lapping plate and polishing pad. For the past few decades, the thermal-curable resin has been primarily selected as the bonding agent in the industry. However, the high-energy consumption, byproduct, and environmental issues stimulated the research in developing a more efficient manufacturing process. The advantages of prototyping technology attracted the attempts from researchers to testify to the feasibility of utilizing light-curable resin in the fabrication of abrasive tools [6,7]. The creative idea of the present research was based on the application of ultraviolet-curable resin prototyping technology, which helps us easily developed an abrasive tool with the capability of generating a hybrid material removal mode of two-body and three-body abrasion during the machining process. Compared with the conventional sintering process with thermal-curable resin, this novel technique significantly reduces the curing time and energy cost. Moreover, In order to verify the practical machining performance of this unique ultraviolet-curable resin bond abrasive tool, we conducted a group of comparative experiments on the technical ceramic workpiece, between the conventional iron plate lapping process and the ultraviolet-curable resin bond lapping plate. It is hoped that this study could be undertaken to help guide a new direction of the precision machining technology of hard and brittle materials.

## 2. Materials and Methods

### 2.1. Ultraviolet-Curable Resin

The ultraviolet-curable epoxy resin prepared for this study was supplied by Dymax Corporation (Torrington, CT, USA), labeled as light weld 425 optically clear structural adhesive. From our previous research on the feasibility of using ultraviolet-curable resin as a bonding agent in the manufacturing of abrasive tools [8], we compared the material properties of the cured resin from different vendors. The 425 resin mentioned above showed some favorable advantages over the others. The technical specification of the cured pure 425 resin is listed in Table 1.

### 2.2. Diamond Abrasive Grain

**Figure 1:**
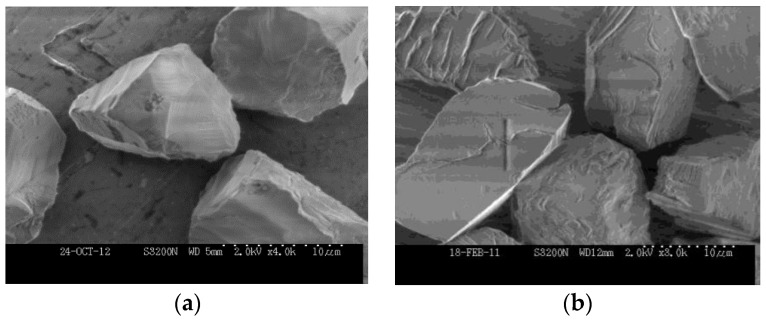
Scanning electronic micrographs of (**a**) the regular monocrystalline diamond grains and (**b**) the surface textured monocrystalline diamond grains.

The abrasive machining tools involved in the machining process of hard and brittle materials mainly utilized with super abrasives as cubic boron nitride (CBN) and artificial synthetic diamond. The CBN tool is mainly used in the machining of the hard metallic material since the diamond is reactive to the ferrous metal at high temperature. Diamond abrasive is superior in hardness, strength, thermal conductivity, and expansion coefficient. It is primarily employed in the machining of hard and brittle material including ceramic, optical glass, and semiconductor material. The abrasive grains selected in this research are the surface textured monocrystalline diamond grains average sized in 15 µm, provided by Engis. Compared with the standard monocrystalline diamond grains, the grain surface of the ones above was textured through a specific etching process, in which the monocrystalline diamond was eroded by oxygen, oxygen compounds, molten metals, and hydrogen at an elevated temperature. As a result, some material on the surface layer of the diamond grains was removed by the erosion and a rough surface was generated. According to our previous study [9,10], the rougher surface of the diamond grain increased the contact surface area between the grains and the bonding agent and consequently improved the bond retention of the abrasive tools. Scanning electronic micrograph comparison of the regular diamond grains and surface textured ones are shown in the figures below, where the surface patterns and pits can be seen in Figure 1b.

### 2.3. Ultraviolet Curing Systems

**Figure 2:**
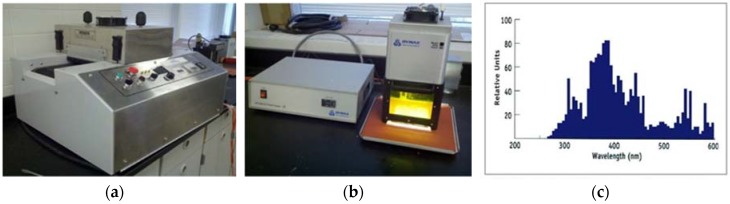
(**a**) Innovative Machine UV-100 ultraviolet curing system; (**b**) Dymax 5000 flood ultraviolet curing system; and (**c**) wavelength distribution of the curing system after optimization.

The ultraviolet curing system for laboratory use mainly consists of an ultraviolet lamp with focalization setup and a power supply. In this study, a customized ultraviolet curing system based on the Innovative Machine UV-100 was primarily employed in the manufacturing experiments of the ultraviolet-curable resin bond abrasive tool, and the Dymax 5000 flood ultraviolet curing system from DYMAX was also used for initial material properties test of the cured ultraviolet-curable resin composites. The curing systems mentioned above are shown in Figure 2. According to the supplier of the resin used in this series of experiments, the recommended wavelengths of the ultraviolet light to initiate the photoreaction is around 365 nm. Hence, the ultraviolet light source of the curing system was professionally optimized to the range shown in Figure 2c. Meanwhile, the detailed technical specification of the curing system is provided in Table 2 below.

### 2.4. Experimental Fabrication Method

**Figure 3:**
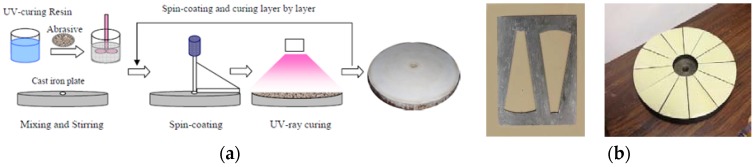
Schematic diagram of the curing processes of the ultraviolet-curable resin bond abrasive tool through (**a**) spin-coating method; and (**b**) fan-shaped pieces assembly method.

In the experimental fabrication of the ultraviolet-curable resin bond abrasive tool, we developed various laboratory methods to realize the manufacturing. Firstly, the ultraviolet-curable resin and diamond abrasives were uniformly mixed through a stirring machine in the darkroom to prevent any unexpected photoreaction. The composite mixture stood in vacuum conditions for 30 min to release the air bubbles generated in the stirring step. After that, the mixture in liquid form can be either spin-coated on top of the base plate or injected into the separated fan-shape mold for the curing process. For the latter method, the cured fan-shaped pieces were going to be arranged and adhered to the base plate to assemble the desired tool. The schematic diagram of the curing processes of the ultraviolet-curable resin bond abrasive tool is illustrated in Figure 3. The spin-coating method was efficient in curing time and it also ensured the integrity of the cured resin plate. However, since the spin-coated layer of resin and abrasives on top of the base plate was dimensionally large, it was difficult to set up an evenly-distributed ultraviolet exposure in practice. As a result, the photosensitive reaction completion differed by the distance from the light source to the surface of the layer, therefore, the cure depth of the layer could be varied to generate a waviness in the resin plate. The dimensional accuracy and machining efficiency can be principally influenced by this fabrication failure. Hence, the fan-shape molding fabrication method was utilized in the curing process in order to assure the ultraviolet energy absorbed and the corresponding photoreaction within a small area is uniform.

In the manufacturing industry of diamond abrasive tools, the diamond concentration is particularly used as a standard to evaluate the weight of diamond in a unit volume of the tool matrix, and it is defined that where each cubic centimeter contains 0.88 g of diamond, the concentration is 100%. In precision machining of hard and brittle materials, a relatively lower abrasive concentration is preferred. On the other hand, the amount of ultraviolet energy passing through the resin and diamond composite strongly depends on the abrasive grain size and concentration, the increase in diamond concentration would decrease the energy absorbed by the sub-surface and bottom layer of the resin composite, and thereby decrease the cure depth of the resin plate [11,12]. For this reason, the diamond abrasive concentration selected in this study is 12.5% to satisfy the requirement of the cure depth, which indicates that each cubic centimeter of the cured resin matrix contains 0.11 g of diamond grains.

### 2.5. Experimental Machining Test

A group of comparative machining performance test was conducted between the conventional slurry-involved lapping process and the process with the ultraviolet-curable resin bond abrasive tool. Technical ceramic ring-shaped samples from Nanjing Co-Energy Optical Crystal Co. Ltd. (Nanjing, Jiangsu, China). were selected as the workpiece with an initial surface roughness of 0.45 µm, some of the material properties of the workpieces can be found in Table 3 below.

**Figure 4:**
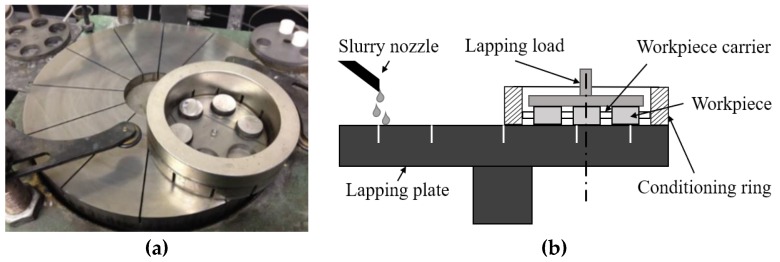
(**a**) The experimental setup and (**b**) schematic diagram of the machining performance test on ceramic workpieces.

Each of the workpieces was cleaned through ultrasonic washing and left until dry, the weight of each workpiece was examined by an electronic balance. A photograph with the schematic diagram of the machining test setup is shown in Figure 4, on which six ceramic workpieces were machined at one time. During the machining test, the workpieces were removed and ultrasonically cleaned in acetone every 10 min to record the surface roughness and weight loss. The surface roughness was measured by a Zygo optical profiler, where a 10× magnification Mirau interference objective was equipped. In the surface roughness measurement, filtering is used to highlight the roughness (high-frequency, short-wavelength component) or waviness (low-frequency, long-wavelength component) of a test part. The filtering method in the Zygo optical profiler employed in this series of measurements was set to low pass with a specified wavelength of the higher cutoff point at 5.47 µm. The surface topography was examined with an atomic force microscope. To maintain the consistency of the experiments, the roughness parameter R_a_ was measured from three randomly-selected areas of 0.70 mm by 0.52 mm on the surface of the machined workpiece. The parameter was based on a pre-positioned straight line that crossed the selected area in each measurement. An average was taken on the roughness parameter R_a_ from the six workpieces periodically, and the weight loss of each workpiece was evaluated on an electronic balance to study the material removal efficiency of the respective machining process. The process parameters of the machining test on the ceramic workpiece are listed in Table 4. The 15 µm diamond grain slurry used in the iron plate lapping was purchased from Engis in standard concentration, that is, 400 g of diamond abrasive per 750 mL, according to their technical specifications.

## 3. Results and Discussion

**Figure 5:**
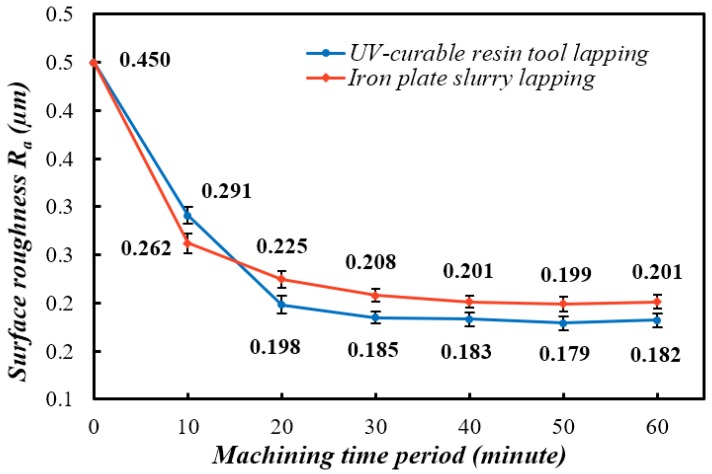
The surface roughness of the ceramic workpiece machined with iron plate lapping and ultraviolet-curable resin tool.

Figure 5 shows the average roughness parameters periodically recorded from both the machining process of conventional lapping on an iron plate and fixed abrasive lapping on the ultraviolet-curable resin bond tool. The lowest roughness parameter R_a_ achieved by iron plate lapping and ultraviolet-curable resin plate lapping is 0.201 µm and 0.182 µm, respectively. In most cases, the machined surface roughness is mainly influenced by the grain size of the abrasives. However, considering that the diamond abrasives employed either in the fabrication of resin plate or the slurry mixture were from the same batch in this study, the differences between those two machining processes could be possibly explained with their abrasive wear mechanisms in material removal.

**Figure 6:**
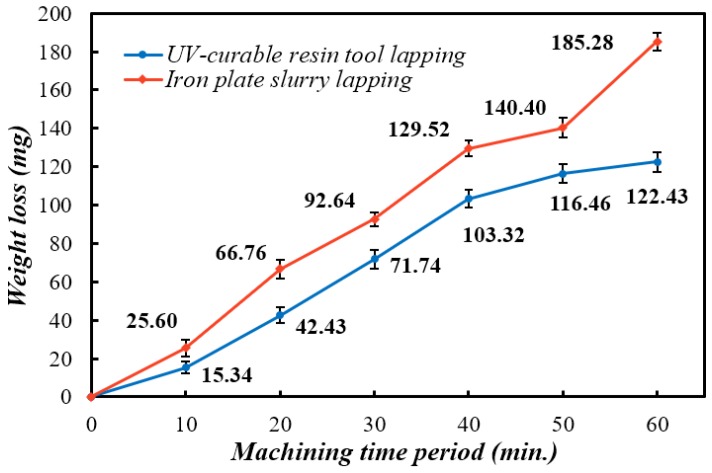
Periodical weight loss of the workpieces.

In the beginning 10 min of the machining process, the surface roughness measured from the ceramic workpiece machined with the iron plate is 0.26 µm, which is about 12% lower than 0.29 µm in resin plate lapping. This phenomenon could be explained by the different material properties of the plates. In the case of iron plate lapping, the diamond abrasive grains immediately started to work as long as they were introduced by the slurry and spread in the working zone between workpieces and the iron plate. Slurry-based loose abrasive lapping is a typical process of the three-body abrasion mode, and it is usually considered as a lower-efficiency process to remove material than two-body abrasion lapping. According to Rabinowicz et al. [13], the abrasive grains in three-body abrasion spend 90% of their working time rolling and performing low material removal rate, that can be ten times higher in two-body abrasion [5]. In our ultraviolet-curable resin bond lapping plate, the diamond grains were uniformly distributed and fixed within the tool. Ideally, they should work in two-body abrasion mode as the other fixed abrasive lapping plate, and thereby produced a higher material removal rate. However, because of the gravity settling of the diamond grains in the resin during the curing process, most of the abrasives were buried within the cured resin matrix. At the beginning of the machining process, only a small number of initial protruding diamond grains worked in two-body abrasion mode to remove material from the workpiece. Therefore, the removal rate is relatively lower than that in iron plate lapping. Hence, the volume of material removed is not enough to degrade the surface roughness parameter R_a_ to the level achieved in iron plate lapping. This assumption also matches the periodical weight loss of the workpieces in the first 10 min, shown in Figure 6.

**Figure 7:**
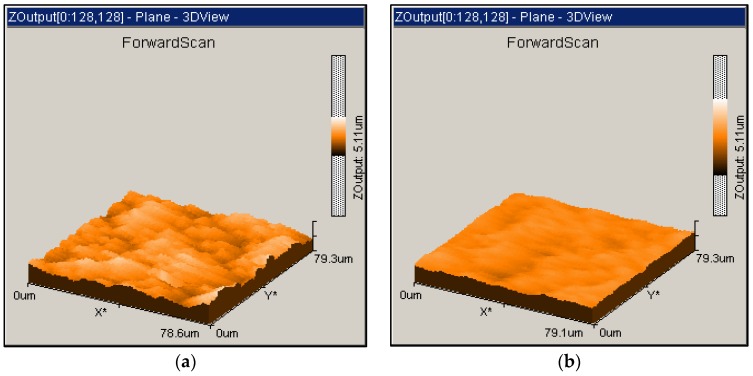
Surface topographies of a ceramic workpiece machined with (**a**) iron plate slurry lapping; and (**b**) ultraviolet-curable resin bond tool lapping.

After 10 min in Figure 5, the surface roughness downward trend is gradually becoming weaker in both processes. The parameter R_a_ from ultraviolet resin tool lapping surpasses that in iron plate slurry lapping between 10 to 20 min by achieving 0.198 µm. Until the end of the machining test, the periodical measured surface roughness of the resin tool lapping remains at the lower state than the iron plate lapping. As is known, the bonding agent of cured ultraviolet-curable resin is weaker in material properties in terms of hardness, strength, and wear resistance, especially when compared to the metallic or vitrified bond. As a result, some of the protruding grains or fall off grains might be pressed into the resin matrix due to its plastic deformation under the lapping load. This phenomenon would effectively decrease the abrasive grain size and thereby cause disadvantages in machining efficiency. However, from another point of view, the decreased abrasive size and soft bonding matrix would also decrease the machined surface roughness and cause less surface damage. Additionally, the featured viscoelasticity of the resin matrix is considered to help to reduce the surface damage of the workpiece, and improve the process consistency and stability [14]. Thus, in this experiment, a better surface quality could be obtained through the ultraviolet-curable resin plate than the iron plate. This hypothesis reasonably explains the surface roughness difference in Figure 5 and the surface topographies comparison in Figure 7. The relatively rougher surface profile in Figure 7a is a small area of 78.6 µm by 79.3 µm from one of the machined workpieces after 60 min lapping with the iron plate, and the smoother one in Figure 7b is from the resin plate lapping after 60 min.

It is noted that, in Figure 6, the material removed in the iron plate slurry lapping is 185.28 mg in total after 60 min, while the number in ultraviolet-curable resin tool lapping is 122.43 mg. It is nearly a 51.34% dropdown in machining efficiency. Additionally, the weight loss trend of the two processes is also quite different. Due to the delivering of lapping slurry, fresh and sharp diamond grains were continuously introduced to the working zone. Hence, the slope rate of the iron plate slurry lapping is constant and varies within a reasonable range. In the case of the resin bond plate, it is assumed that all the diamond grains were embedded in the cured resin matrix of the abrasive tool and worked as a fixed abrasive grain at the beginning. As the machining process continues, the resin matrix started to wear out due to the low hardness and wear resistance. According to the studies in diamond retention of abrasive tools [15,16], the abrasive grains are largely held within the bonding matrix by the mechanical compression generated during the manufacturing process. Therefore, the retention force is predominately determined by the material properties of the solidified bonding agent, which is the ultraviolet-curable resin in this case, considering the disadvantages of cured resin in material properties regarding strength and hardness, within which the diamond abrasive grains tend to be pulled out from the bonding matrix with ease than metallic or vitrified bonding.

Hence, some of the initially-fixed abrasive grains began to fall off from the bonding matrix and turned into loose abrasive grains working in three-body abrasion. Meanwhile, the abrasive grains buried in the underlayer of the resin matrix was continuously revealed and worked as fixed abrasive grain renewedly. This unique mechanism provides the resin tool lapping with fresh diamond grains like the slurry does in iron plate lapping. Additionally, the probability of the falling off diamond grains being pressed into the resin plate exists and converts the loose abrasive grains into fixed ones. Because of this, the lapping process of the ultraviolet-resin bond plate can be summarized as a hybrid process in which the two-body abrasion and three-body abrasion material removal mode corporately contribute to the machining process, while the interconversion between fixed grains and loose grains could be dynamically balanced at a certain period of the process. This presumption explains the stabilization of surface roughness in the resin tool lapping process after 40 min.

According to the data in Figure 6, the weight loss of the machined workpiece in resin tool lapping also becomes stable after 40 min. That means the material removed from the workpiece after 40 min is rapidly reduced. The weight loss of the workpiece in the first 40 min is 103.32 mg, while the number in the period from 40 to 60 min is 19.11 mg. Since the ultraviolet-curable resin bond lapping plate was fabricated in the laboratory, the manufacturing process flaws would cause some of the failures in machining performance. For instance, the absence of diamond grains at certain layers of the tool due to the non-uniform distribution of abrasives, the unstable hardness of the fabricated resin plate because of the incomplete photoreaction during the curing process, and the glazing issue caused by the dull grain and porosity stuck.

**Figure 8:**
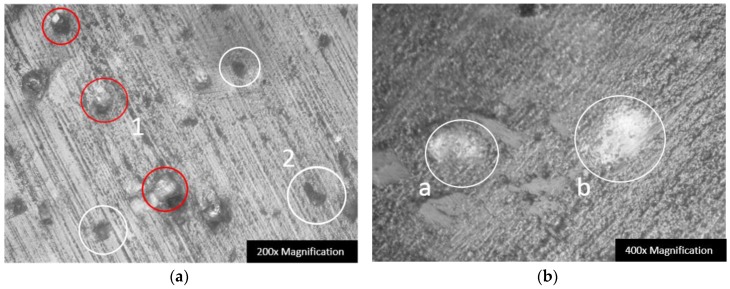
Worn surface of the ultraviolet-curable resin plate after lapping process: (**a**) the diamond grains working condition at 200× magnification with the active protruding diamond grains circled in red and pull-out holes circled in white, and (**b**) the diamond grains embedded within the resin matrix at various underneath heights circled and labelled as *a* and *b*.

An optical image observation on the worn surface of the ultraviolet-curable resin plate after the lapping process is shown in Figure 8. As we presumed, the number of the pull-out holes, which are marked with white circles in Figure 8a, indicate the weaker strength and retention force of the resin matrix to hold the diamond grains from being pulled out. The protruding diamond grains marked in red circles are the fixed abrasives that are primarily employed to remove the materials in the two-body wear mode during machining, while the three-body working abrasives were rolling between the workpiece and the tool and continuously carried away with the machining coolant. Moreover, the nonuniform distribution of the fixed diamond grains and pull-out holes on the worn surface of the tool in Figure 8a reflects the fabrication disadvantage in the laboratory. A 400× magnification on the worn surface in Figure 8b clearly shows the diamond grains that are embedded within the resin matrix after lapping, and it refers to the fact that, as the machining process continues, the fresh diamond grains are not ensured to reveal with the resin matrix wear. In practice, all these potential defects mentioned above possibly lead to the failure of the tool’s machining capability. Thus, the improvement in manufacturing process optimization and material selection could be directions for future work.

## 4. Conclusions

This study proposed a new type of fixed abrasive lapping plate for precision machining on hard and brittle materials, in which the ultraviolet-curable resin was selected as the bonding agent in the fabrication of the abrasive tool to deliver a rapid, flexible, economical, and environment-friendly manufacturing process. The performance of the ultraviolet-resin bond diamond lapping plate was examined through the comparative experiments with slurry-based iron plate lapping. The conclusions can be summarized as follows:

- The fabrication process of the ultraviolet-curable resin bond plate was completed within a minute light exposure, saving energy costs and labor effort, which occurs in the conventional sintering process.
- In the ceramic workpiece lapping process, the ultraviolet-curable resin bond lapping plate was enabled to achieve an approximately 10% lower surface roughness parameter R_a_ than that in the iron plate lapping process.
- In the study of the material removal rate evaluated by weight loss of the workpiece, the resin plate lapping showed about 25% less material removed per minute in the stable machining state.
- The machining performance of the resin plate can be explained by the hypothesized discussion that an integrated abrasion mode of two-body and three-body wear was established.

However, the study on the control of the fabrication process parameters that affect the material properties of the cured resin matrix, the methodology to obtain a uniform distribution of the abrasive grains within the bonding agent, and the appropriate technique to evaluate the working condition of the tool is still limited in this research. The effort on these directions should be taken in the future studies regarding related works. It is hoped that this experimental study could inspire the application of the ultraviolet-curable resin bond abrasive tool, and ultimately integrate the two-step precision flattening process of lapping and polishing into one.

## Figures and Tables

**Table 1:** Technical specification of the ultraviolet-curable resin used.

Material Properties	Density (g/mL)	Viscosity cP (20 rpm)	Hardness (Durometer)	Tensile (psi)	Elongation (%)	Modulus of Elasticity (psi)
Dymax 425	1.07	4000	D80	6200	7.3	500,000

**Table 2:** Technique specifications of the ultraviolet curing system.

Output Power	Ultraviolet Light Source	Typical Intensity (320–390 nm, 7.62 cm from the Bottom)	Illumination Area
400 Watts	UVA ^1^ flood	225 mW/cm^2^	161.29 cm^2^

^1^ Wavelengths of the ultraviolet are classified as UVA, UVB, or UVC, with UVA at 320–400 nm.

**Table 3:** Material properties of the ceramic workpiece.

Chemical Formula	Density	Hardness	Tensile Strength	Modulus of Elasticity	Poisson’s Ratio
96% Al_2_O_3_	3.65 g/cm^3^	85 HRA	160 MPa	300 GPa	0.20

**Table 4:** Process parameters of machining tests on the ceramic workpiece.

Lapping Machine	Diameter of Lapping Plate	Diameter of Workpiece Carrier	Minimum Rotation Speed	Maximum Rotation Speed	Round Count Precision	Lapping Pressure	Lapping Period
Lap Master 12	30.5 cm	10.8 cm	5 RPM	50 RPM	±0.5°	1.77 KPa	10 min
